# Extraction, Characterization, and Evaluation of *Lepidium sativum* Linn. Mucilage as a Mucoadhesive Polymer

**DOI:** 10.1155/2023/5535344

**Published:** 2023-08-14

**Authors:** Hailemichael Embafrash Berhe, Desta Tesfay Mezgebo, Solomon Abrha, Tsadkan Gebremeskel Haile, Fantahun Molla

**Affiliations:** ^1^Department of Pharmacy, Ayder Comprehensive and Specialized Hospital, College of Health Sciences, Mekelle University, P.O. Box 1871, Mekelle, Ethiopia; ^2^Department of Pharmaceutics, School of Pharmacy, College of Health Sciences, Mekelle University, P.O. Box 1871, Mekelle, Ethiopia; ^3^Department of Pharmaceutics and Social Pharmacy, School of Pharmacy, College of Health Sciences, Addis Ababa University, P.O. Box 1176, Addis Ababa, Ethiopia

## Abstract

Being biocompatible, less toxic, cheap, easily available, and environmentally friendly, there is an increased trust in natural polymers in the drug delivery system. Mucilages, among the natural polymers, are the primary metabolites of plants that have been widely utilized in pharmaceutical manufacturing for different purposes, and mucoadhesive is one among them. The present study was designed to investigate the use of LSM as a mucoadhesive polymer using ibuprofen as a model drug. The mucilage was extracted following an aqueous extraction method and its percentage yield was found to be 13.2% w/w. Besides, three microsphere formulations of ibuprofen were prepared using synthetic polymer hydroxyl propyl methyl cellulose (HPMC) K100M and the LSM in polymer to drug ratios of 1 : 1, 1 : 5, and 3 : 5 by applying ionotropic gelation followed by solvent evaporation methods. The microspheres were evaluated for various micromeritic properties and all the formulations exhibited free-flowing properties. Optical microscopic pictures of almost all the microspheres except F3 and F6 (which had more or less spherical shapes) were found to have irregular and discrete shapes. Besides, the surfaces of all the formulations were rough in texture. The drug entrapment efficiency of the microspheres was found to be between 52.08% ± 0.80 and 87.97% ± 0.72. The *in-vitro*wash-off test evidenced that almost 50 percent (especially F3) of the microspheres were able to adhere up to 18 h and showed remarkable bioadhesion properties. The *in-vitro* drug release profile indicated that all the formulations were able to prolong their drug release up to 12 h with a non-fickian release mechanism, except for F4, which followed a fickian release. Therefore, based on the findings of this study, LSM can be used as a potential alternative mucoadhesive excipient for sustained release formulations.

## 1. Introduction

The oral route remains the most convenient route of drug administration due to its ease of administration, patient acceptability, less stringent production conditions, and low cost [1]. Despite the potential advantages, majorities of pharmaceutical formulations delivered through the oral route have numerous challenges pertaining to bioavailability [2]. Besides, the bioavailability of oral formulations is affected by the molecular weight, hydrophobicity, hydrophilicity, and inadequacy of physicochemical and biopharmaceutical properties such as solubility, stability, permeability, and metabolic stability of the drug [2].

Mucoadhesive drug delivery systems (MDDS) are being explored for the localization of the active agents to a particular site [3]. Moreover, studies conducted to solve the challenges pertaining to oral bioavailability have shown that microspheres can improve the bioavailability of drugs through mechanisms such as mucoadhesion. The term mucoadhesion is often employed when the biological substrate is a mucosal surface [4, 5]. Likewise, in the gastrointestinal tract, mucoadhesion is the adhesion of semisolid forms to the mucus. Furthermore, mucins play the key role in maintaining the gel-like properties of the substrate for the potential binding of polymers for site-specific drug delivery [6]. The rationale for developing a mucoadhesive drug delivery system lies behind the fact that the formulation could be held on a biological surface for localized drug delivery. Active pharmaceutical ingredients (APIs) can be released close to the site of action, with a consequent enhancement of bioavailability [7].

Polymers play an important role in designing mucoadhesive drug delivery systems via increasing the residence time of the active agent at the desired location [3]. Swelling of the polymer induces a mechanical entanglement, which exposes the bioadhesive sites to hydrogen bonding and/or electrostatic interaction between the polymer and the mucous network [8]. The swelling property of polymers is primarily associated with the substituted groups of the polymer such as hydroxyl groups which plays a remarkable role in the integrity of the matrix which in turn is determined by the amount and properties of the incorporated drug [6]. Different synthetic polymers such as polyacrylic acid and its derivatives, hydroxyl propyl methyl cellulose and various grades of carbopol have been investigated for their mucoadhesion characteristics [9]. However, synthetic polymers are accompanied with a drawback related to being expensive in cost and they have a problem with their biodegradability and biocompatibility property [10].

In recent years, naturally derived polymers such as sodium alginate, chitosan, starch, aloe vera, *Ceratonia siliqua*, *Opuntia ficus indica*, *Basella alba,* and *Lepidium sativum* Linn. have received tremendous interest as potential excipients for pharmaceutical preparations. Moreover, among the various constituents of plants, mucilage has been preferred owing to its biocompatibility, ease of availability, lack of toxicity, low cost, soothing action, and nonirritant nature [10, 11]. Mucilages are abundant in nature and commonly found in rhizomes, roots, and seed endosperms of many higher plants. Because of the high concentration of hydroxyl groups, mucilages generally have a high-water binding capacity and swell very perceptibly in water [12].

**Figure 1:**
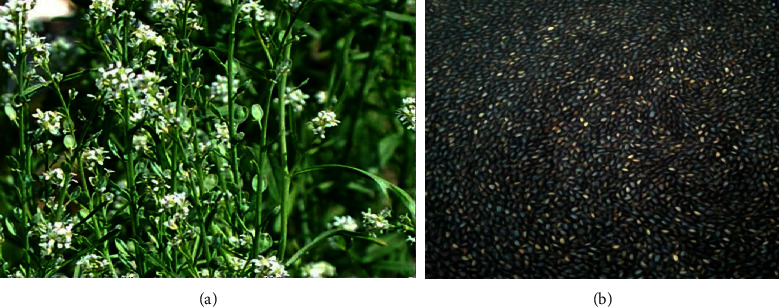
*Lepidium sativum* Linn. plant (a) and *Lepidium sativum* Linn. seed (b) (picture taken by Hailemichael. E).

*Lepidium sativum* Linn., an annual herb (Figure 1(a)), belongs to the family of *Brassicaceae*. Its distribution is limited to the mountainous and alpine regions in the tropics of East Africa such as Kenya, Tanzania, and Ethiopia [13, 14]. It is a polymorphous species and is believed to have originated primarily in the highland regions of Ethiopia and Eritrea [15, 16]. In Ethiopia, *Lepidium sativum* Linn. (known as *Fetto* in Amharic and *Shinfa'e* in Tigrigna) is widely used as a traditional medicine. It is a fast-growing edible annual that can be cultivated at any season which can be evidenced by the availability of its seeds (Figure 1(b)) in local markets at a very low cost [16].

It is made of nonstarch polysaccharides (90%) and starch (10%) [17]. The chemical compositions of *Lepidium sativum* seed (LSS) polysaccharides include the major sugars of mannose (38.9%), arabinose (19.4%), galacturonic acid (8%), fructose (6.8%), glucuronic acid (6.7%), galactose (4.7%), rhamnose (1.9%), glucose (1%), and some uronic acid (15%) which are the characteristic compositions of a mucilage. Besides, the ratio of mannose to galactose is 8.2. Thus, in the presence of water, the polyuronide chains containing ionizable carboxyl groups become hydrated and swell and the cellulose micelles become dispersed. Moreover, the seed bran (coat) has a high dietary fiber content and it has a high-water holding capacity (74.3%) [18, 19].

As a result of the wide pharmaceutical applications of mucilages, plants containing mucilages have been recently given due attention. Ethiopia is well endowed with a variety of mucilage-bearing plant sources, which can be explored and utilized as pharmaceutical excipients [13]. So far, mucilages of different plant species have been explored for their pharmaceutical application but a study on the mucoadhesive property of *Lepidium sativum* Linn. mucilage (LSM) is lacking. Thus, the present study was aimed at evaluating LSM for its mucoadhesive properties in ibuprofen microspheres.

## 2. Materials and Methods

### 2.1. Materials, Chemicals, and Reagents

Dried seeds of *Lepidium sativum* Linn. were procured from the local market of Saesie kebele, Saesie Tsaeda Emba district, Eastern zone of Tigray, Ethiopia and identified by Dr. Yemane Gebrezgabher, a botanist in Mekelle University, and the plant was authenticated by the Department of National Herbarium, Addis Ababa University. Ibuprofen (Batch no. C100-1511146M, Universal Company, China) and HPMC K100M (Batch no. 140102, Anhui Sunhere Pharmaceutical Excipients Co. Ltd, China) were donated by the Addis Pharmaceutical Factory P.L.C (APF). Acetone (Batch no. V3E490203F, Carlo Erba, France), hydrochloric acid BP (Batch no. V1N797131N, Carlo Erba, France), disodium hydrogen orthophosphate (Batch no. C6F8CP01, Loba Chemie, India), potassium dihydrogen orthophosphate (Batch no. 7778-77-0, Loba Chemie, India), dichloromethane (Batch no. 4422AR, Blulux Laboratory, India), and distilled water were purchased from local markets. All the chemicals and reagents were of analytical grade and used as received.

### 2.2. Methods

#### 2.2.1. Extraction of Mucilage from *Lepidium sativum* Linn. Seed

Initially, dried seeds of *Lepidium sativum* Linn. were screened for any physical or mechanical damage.

They were then washed with distilled water. One hundred gram of the dried seeds was soaked in 800 ml of distilled water for 12 h. Then, the soaked seeds were blended (Blender 8011EG, Model HGB2, serial number 677) at 2000 rpm for 15 min.

The blended seeds were filtered through a muslin cloth. Then, an additional 200 ml of distilled water was added to the seeds and again blended and refiltered through the muslin cloth (repeated twice). Afterwards, an equivalent amount of acetone was added to the filtrate and the mucilage was allowed to precipitate. The precipitated mass was then separated through filtration using a muslin cloth. Precipitated mucilage was dried in an oven (Genlab Limited Y4C004, England) at a temperature of 45°C for 16 h. Lastly, the size of the dried and collected mucilage was reduced using a mortar and pestle, sieved using a sieve number 80 and weighed to calculate the percentage yield, and then transferred to a closed container [20].

#### 2.2.2. Characterization of LSM

*(1) Phytochemical Screening of LSM*. The LSM was characterized against the different phytochemical constituents. Appropriate testing procedures were applied to screen and verify the presence or absence of constituents such as proteins, tannin, alkaloids, saponins, and carbohydrates in the extracted mucilage [18, 21].

*(2) Loss on Drying (LOD)*. Five grams of the mucilage was transferred into cleaned Petri dishes and dried in an oven (Genlab Limited, serial no. Y4C010, England) at a temperature of 105°C for 24 h until a constant weight was obtained. The percentage loss of moisture on drying was calculated using equation (1) [22].

(1) $$\begin{array}{c} Loss\;on\;drying\;\left(\%\right)=\frac{Initial\;weight-Final\;weight}{Initial\;weight}\times 100\text{.} \end{array}$$

*(3) Particle Size*. The particle size of the dried powder of mucilage was determined using the sieve analysis method. Accordingly, 10 g of the sample was tested for particle size distribution after sieving the sample for 5 min (USP 2013).

*(4) pH of Solution*. pH of a 0.5% (w/v) solution of mucilage in distilled water was determined using the pH meter (Adwa, Serial no. AD8000, Hungary) [18].

*(5) Swelling Index (SI %)*. The swelling ratio of the mucilage was measured by first preparing 100 ml of distilled water, 0.1 N hydrochloric acid (HCL) of pH 1.2, and phosphate buffer solution (PBS) of pH 6.8 using a stoppered graduated cylinder. Then, one gram of the dried mucilage was added to different empty cylinders and the initial bulk volumes of the mucilage were recorded. The aforementioned three medias were then added in sufficient quantity to yield a 100 ml of uniform dispersion. The dispersions were stored at room temperature and the weights of the swollen masses were measured after 24 h [18, 23]. The swelling index was calculated by using the following equation:

(2) $$\begin{array}{c} Swelling\;Index\;\left(SI\;\%\right)=\frac{Wt-Wo}{Wo}\times 100\text{,} \end{array}$$

where Wt = weight of swollen mucilage at time *t* and Wo = initial weight of mucilage.

*(6) Flow Property*. Thirty grams of mucilage was transferred into a 250 ml measuring cylinder. The density and density-related properties such as bulk and tapped densities, Carr's index, and Hausner's ratio of the mucilage were determined using the established standard methods [22]. The angle of repose and the flow rate of the mucilage were measured on 50 g of the mucilage powder using an automated powder flow analyzer (PTG-S4, Pharma Test, Germany) [22].

*(7) Viscosity Determination*. The viscosity of the mucilage was measured by preparing different concentrations of the mucilage (0.1–0.5% w/v) in distilled water. The viscosities were measured using a viscometer (Brookfield digital viscometer Model DV-E, USA).

*(8) Drug-Mucilage Compatibility Study*. Drug-mucilage interaction was studied using Fourier-transform infrared (FTIR) spectroscopy . The FTIR spectra of the pure Ibuprofen, LSM, and equal ratio of the physical mixture of both the drug and mucilage samples were scanned at room temperature using an FTIR spectrophotometer (FTIR- A21004401166LP, Shimadzu, Japan). The samples were ground in a mortar and pestle and 8 mg of the finely ground samples were mixed with an oily mulling agent (Nujol) in a mortar and pestle. The sample mixtures were then placed on a potassium bromide (KBr) plate and a second plate was placed on top of the first salt plate and compressed to form a thin film of the mull. The sandwiched plates were placed in the FTIR spectrometer to obtain the spectra. All the FTIR spectrums were collected with four scans and a resolution of 2 cm^−1^. Spectra's were recorded in the range between 4000 and 400 cm^−1^.

#### 2.2.3. Preparation of LSM Microspheres

Ibuprofen mucoadhesive microspheres were prepared from LSM and standard polymer HPMC K100M following the ionotropic gelation technique. In this, calcium chloride was used as a mild cross-linking agent. To prepare different concentrations of formulations of LSM and drug (Table 1) with a ratio of 1 : 5, 3 : 5, and 1 : 1, LSM was dissolved in distilled water. Then, the model drug (ibuprofen) was added to the LSM solution and mixed at 8000 rpm for 2 min using a homogenizer (Kalibra, Technisch Service: 015-2780162, Europe). After that, the dispersion was added dropwise to a 5% (w/v) calcium chloride solution using a 24G needle (kept at a distance of 6 cm from the level of the solution) with continuous stirring at 200 rpm using a magnetic stirrer. The stirring was continued for 30 min to complete the reaction. After 30 min, microspheres were collected, washed with distilled water, and dried overnight at 25°C [23].

#### 2.2.4. Evaluation of LSM Microspheres

*(1) Particle Size and Shape*. Particle size and size distributions of microspheres were measured using the sieve analysis method. Microspheres were separated into different size fractions or percentage mass fractions by sieving for 5 min using standard sieves (Serial no. 03.802/02730, Fritsch, Germany) with nominal mesh size apertures of 1.4, 1.0, 0.71, 0.5, and 0.355 mm. Then, the microspheres retained on each sieve were collected separately and weighed and calculated using equation (3) [12, 22]. On the other hand, shapes of the microsphere formulations were observed using an optical microscope (Serial No. 445900, Optika, Italy) with a micrometer scale and a calibrated ocular micrometer at maximum possible resolution (eyepiece 10x and magnification 40) [24].

(3) $$\begin{array}{c} Mean\;particle\;size=\frac{\sum \left(Mean\;particle\;size\;of\;the\;fraction\;x\;Weight\;fraction\right)}{\sum \left(Weight\;fraction\right)}\text{.} \end{array}$$

*(2) Density and Density-Related Properties*. Microspheres were evaluated for the bulk and tapped densities, Carr's index, Hausner's ratio, and angle of repose by using the same methods described above. Accordingly, 30 g of sample microspheres was transferred into a 250 ml measuring cylinder. Then, the volume occupied by the sample was noted and bulk volume was calculated. For tapped density, the volume of the microsphere was recorded after manually tapping the cylinder 250 times. Besides, the compressibility index (CI) and Hausner ratio were calculated from the bulk and tapped densities [12].

*(3) Angle of Repose*. To measure the angle of repose, 50 g of microspheres were made to flow through a stainless steel funnel with an internal diameter of 10 mm using a powder flowability tester (Pharma Test, Germany).

*(4) Drug Content, Entrapment Efficiency, and Percentage Yield*. The entrapment efficiency of the prepared microspheres was determined by extracting the drug present in the microspheres. First, the dried microspheres (100 mg) were taken and extracted in 100 mL of phosphate-buffered saline (PBS) with a pH of 6.8 for 4 hours. Then, the dispersions of microspheres were centrifuged at 3000 rpm for 30 min and filtered through a 0.45 *μ*m filter. Finally, the polymeric debris was washed three times with fresh PBS to extract any adhering drug and the entrapment efficiency was calculated using equation (4) following a standard calibration curve prepared using pure ibuprofen [25, 26]. The drug content of the filtrate was determined at *λ*_max_ of 272 nm using a UV/Vis Spectrophotometer (JENWAY, Model no. 6405, UK) and calculated using equations (5) and (6).

Furthermore, the percentage yield was determined using the formula given in equation (7) [27].

(4) $$\begin{array}{c} \%Drug\;entrapment\;efficiency\;\left(\%DEE\right)=\frac{\left(Drug\;added-Untrapped\;drug\right)}{Theoretical\;Drug\;content\;\left(Drug\;added\right)}\times 100\text{,} \end{array}$$

(5) $$\begin{array}{c} Content\;of\;drug\;released\;\left(mg\right)=\frac{\left(Conc\;.X\;di\;lu\;.factor\;X\;Vol\;.of\;disolution\;medium\right)}{1000}\text{,} \end{array}$$

(6) $$\begin{array}{c} Drug\;released\;\left(\%\right)=\frac{Content\;drug\;released\;\left(mg\right)\times 100}{Dose\;\left(mg\right)}\text{,} \end{array}$$

(7) $$\begin{array}{c} Yield\;\left(\%\right)=\frac{Weight\;of\;dried\;microspheres\;obtained}{Total\;weight\;\left(Drug+Excipients\right)}\times 100\text{.} \end{array}$$

*(5) Swelling Index*. The swelling property of microspheres was determined in PBS (pH 6.8) and in acidic media of pH 1.2 at 37.5°C. Microspheres of each formulation with a known weight of 100 mg were placed in 100 ml dissolution media and kept for 24 h. Afterwards, the microspheres were centrifuged at 1000 rpm to collect the swollen microspheres. Then, the swollen microspheres were initially blotted with a filter paper to remove the absorbed water on the surface and the final weights were determined. Finally, the percentage of the swelling index of microspheres was calculated using the formula mentioned in equation (8) [27].

(8) $$\begin{array}{c} Swelling\;Index\;\left(SI\%\right)=\frac{Wt-Wo}{Wo}\times 100\text{,} \end{array}$$

where Wt = weight of swollen microspheres at time and Wo = initial weight of microspheres.

*(6) Ex-Vivo Mucoadhesion (Wash-Off) Study*. The mucoadhesive property of the microspheres was determined following an *ex-vivo* adhesion testing method known as the *in-vitro*wash-off method by employing the disintegration apparatus (PT ZS Pharma Test, Germany). First, a freshly excised piece of goat intestinal mucosa (3 × 2 cm) was affixed suitably on supported glass slides (3 × 2 cm) with the help of a double-sided cyanoacrylate tape. Then, 100 mg microspheres were spread onto each wet and rinsed tissue specimen, and subsequently hanged onto the arm of a USP tablet disintegration test machine. When the disintegration test machine (PT ZS Pharma Test, Germany) was operated, it was ensured that the tissue specimen was subjected to slow and regular up-down movement in the test fluid of 900 ml physiological solution (PBS of pH 6.8) at 37 ± 0.5°C. An initial count of the number of particles retained on the gut lumen was noted at 30 min, subsequently, counting was made at an interval of 1 h up to 12 h and at 18 h in the buffer media. The machine was switched off and the number of microspheres which remained adhered onto the tissues was counted and calculated using equation (9) [28].

(9) $$\begin{array}{c} Adhesive\;strength\;\left(\%\right)=\frac{Ns}{No}\times 100\text{,} \end{array}$$

where No  = initial number of the microspheres spread over the mucosal surface and Ns = number of microspheres remaining attached with the mucosal surface.

*(7) In-Vitro Drug Release Test*. *In-vitro* drug release of ibuprofen microspheres was studied using the USP paddle-type dissolution apparatus (Pharma Test, Germany). Preweighed microspheres containing 100 mg of ibuprofen were taken from each batch and introduced into each beaker with a 900 ml dissolution medium of 0.1 N HCl pH of 1.2 and PBS of pH 6.8 separately at 37°C ± 0.5°C, with a rotation speed of 100 rpm. An aliquot of 5 ml was withdrawn at different predetermined time intervals; 0, 0.5, 1, 2, 3, 4, 5, 6, 7, 8, 9, 10, 11, and 12 h and filtered through a Whatman filter paper with a size of 0.45 *μ*m. Then, the same volume (5 ml) of fresh dissolution medium, kept at the same temperature, was added after each sampling to maintain the sink condition. Finally, the amount of drug dissolved in the dissolution medium was measured using a UV-visible spectrophotometer (JENWAY, Model no. 6405, UK) at *λ*_max_ of 272 nm [29]. The cumulative percentage of drug release from the ibuprofen microspheres was calculated from the standard curve.

*(8) In-Vitro Drug Release Kinetics*. Data obtained from the *in-vitro* release studies were fitted into various kinetic equations to evaluate the rate and mechanism of ibuprofen release from the prepared microspheres and calculated using the following equations [30]:

(10) $$\begin{array}{c} Zero-order\;model:Q=Kt+Qo\text{,} \end{array}$$

where *Q* is the amount of drug released in time *t*, Qo is the start value of *Q*, and *k* is the rate constant.

(11) $$\begin{array}{c} First-order\;model:Q={Qoe}^{-kt}\text{,} \end{array}$$

where *Q* is the amount of drug released in time *t*, Qo is the start value of *Q*, and *k* is the rate constant.

(12) $$\begin{array}{c} Hixson-Crowell\;Model:Q^{1/3}={Q0}^{1/3}-Kt\text{,} \end{array}$$

where *Q* is the amount of drug released in time *t*, Qo is the start value of *Q*, and *k* is the rate constant.

(13) $$\begin{array}{c} Higuchi\;Modell:Q={Kt}^{1/2}\text{,} \end{array}$$

where *Q* is the amount of drug released in time *t* and *k* is the rate constant.

(14) $$\begin{array}{c} Korsmeyer-Peppas\;Model:Q={Kt}^{n}\text{,} \end{array}$$

where *Q* is the amount drug released in time *t*, *k* is the rate constant, and *n* is the diffusion exponent.

These models were compared based on their accuracy and model prediction capability using the correlation coefficient (*R*^2^). Based on the “*n*” value, the *in-vitro* drug release mechanisms of the formulations were analyzed. Thus, if the value of “*n*” for spherical formulations is < 0.43, it is quasi-Fickian and 0.43 is for Fickian diffusion (diffusion controlled release), “*n*” between 0.43 < *n* ≤ 0.85, is a non-Fickian release (anomalous transport or diffusion), and if “*n*” > 0.85, it is a case-II transport [31, 32].

### 2.3. Statistical Analysis

Statistical analyses of all the data were performed using SPSS software version 21. One-way analysis of variance (ANOVA) was applied for comparison of all results. Moreover, MS Excel, Kinet DS 3.0 Rev.2010, and Origin 8 software were used to draw figures and to determine the cumulative percentage drug release profiles. At 95% confidence interval, *P* values less than or equal to 0.05 was considered significant and all the values are presented as mean and standard deviations.

## 3. Results and Discussion

### 3.1. Phytochemical Screening and Percentage Yield

The yield value of the aqueous solvent extract of LSM was found to be the highest with 13.2% (w/w) on a dry weight basis. In comparison to other studies, the percentage yield of this study was better [19, 32]. Preliminary phytochemical screening of LSM demonstrated the presence of constituents such as alkaloids, carbohydrates, glycosides, flavonoids, proteins, and amino acids (Table 2).

### 3.2. Physicochemical Properties of LSM

#### 3.2.1. Rheological Property

**Figure 2:**
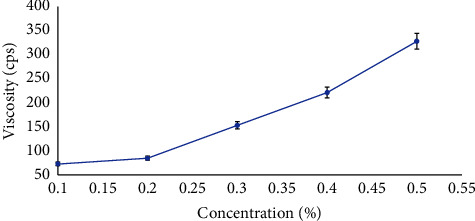
Effect of concentration on the viscosity of dried LSM (*P* < 0.05).

As shown in Figure 2, it was observed that the viscosity of LSM increased with an increase in the concentration of the mucilage. Besides, a maximum viscosity of 328 ± 1.3 cps was obtained at 0.5% (w/v) concentration of mucilage which indicates the extremely viscous characteristics of the mucilage at low concentration. Thus, this property illustrated that LSM can be used as a potential mucoadhesive agent [19].

#### 3.2.2. Moisture Content and Particle Size

The result for loss on drying indicated the presence of 8% of moisture in the extract which is within the pharmacopoeial specification (maximum of 15%) set for mucilage [22]. The mean particle size of the dried mucilage was observed to be 182 ± 1.2 *μ*m (Table 3).

#### 3.2.3. Swelling Index

The swelling index of the LSM in 0.1 N HCl of pH 1.2, PBS of pH 6.8, and distilled water was found to be 2.9 ± 0.2, 16.5 ± 0.3, and 18.8 ± 0.2, respectively (Table 4). Likewise, the swelling index of the mucilage was higher in water followed by PBS and acidic solution, which could be attributed to the mucoadhesive property of the mucilage. Moreover, the swelling ability of the mucilage is associated with the generation of sufficient macromolecular mesh and the liberation of its chains in order to increase the interpenetration between the polymer and mucin [33].

#### 3.2.4. pH

pH is one of the important parameters that determine the suitability of any excipient for a given formulation. Likewise, the physiological activity and stability of most preparations depend on pH [24]. As shown in Table 4, the pH of the LSM solution which was measured at room temperature was found to be 6.2. Hence, this revealed that the pH of the mucilage was nearly neutral which in turn implied the biocompatibility of the mucilage. Thus, formulations prepared using this material would be biocompatible with minor or no irritation to the mucosal membrane of the GIT [34].

#### 3.2.5. Flow Properties

The values of density were used to determine the Hausner ratios and Carr's indices which are the measures of the flowability and compressibility of a powder. Carr's index and the Hausner ratio values of the mucilage were 9.98% and 1.05, respectively, indicating the excellent flow and compressibility properties (Table 3) [22].

#### 3.2.6. Drug-Mucilage Compatibility

**Figure 3:**
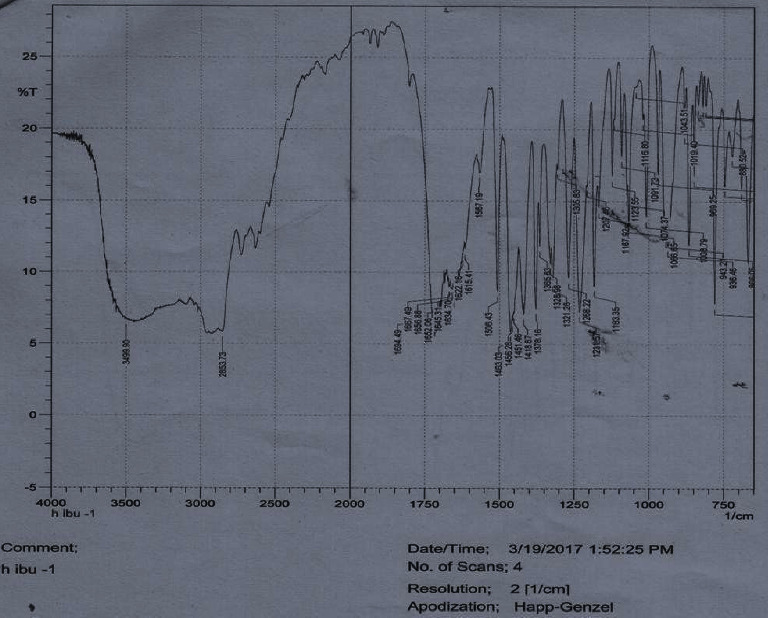
FTIR spectrum of pure ibuprofen.

**Figure 4:**
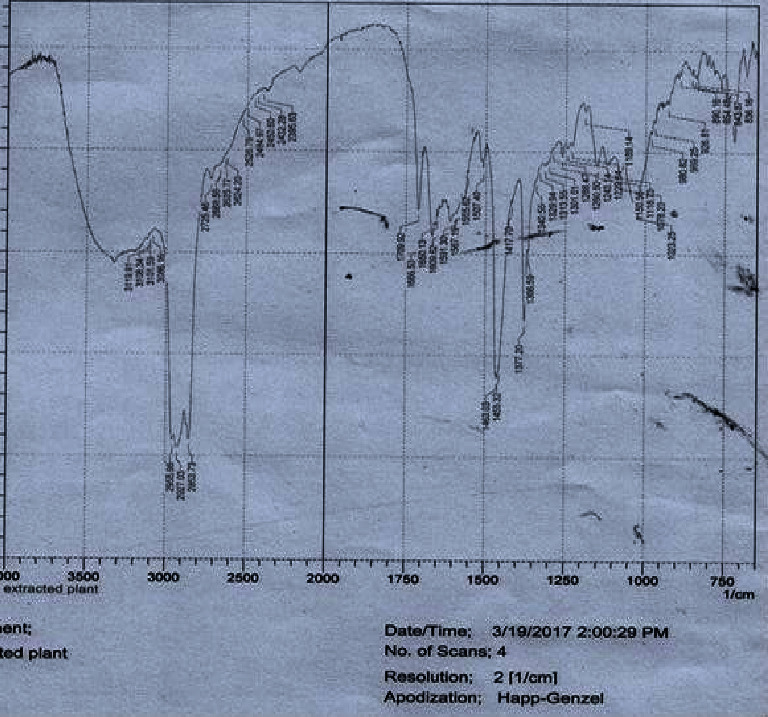
FTIR spectrum of LSM.

**Figure 5:**
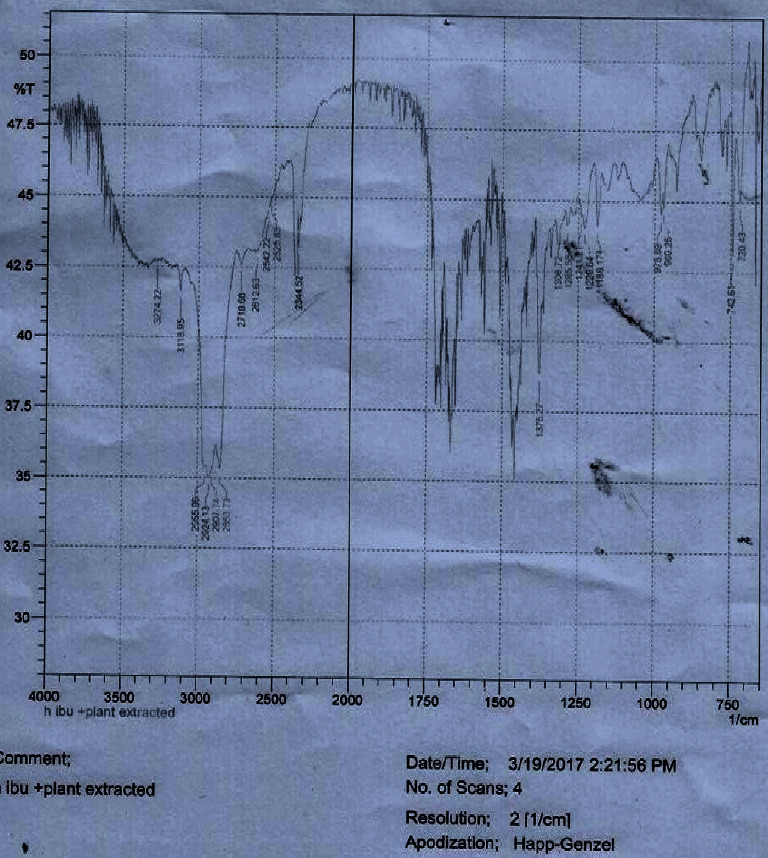
FTIR spectrum of pure ibuprofen and LSM mixture.

FTIR spectroscopy of pure ibuprofen, LSM, and their mixture are shown in Figures 3–5, respectively. The characteristic FTIR absorption peaks of pure ibuprofen are 1463, 1719, 2854, and 3500 cm^−1^ which are shown in Figure 3 [35]. These characteristic peaks of pure ibuprofen (Figure 3) also appeared at similar wave numbers in the spectrum of the physical mixture of ibuprofen and mucilage (Figure 5). The FTIR spectrum of both mixtures neither showed any new absorption spectra nor any change in fingerprint spectra of the drug (i.e., ibuprofen). Thus, this result indicated that there was no incompatibility problem between the drug and the mucilage.

### 3.3. Evaluation of LSM Microspheres

#### 3.3.1. Shape and Morphology

**Figure 6:**
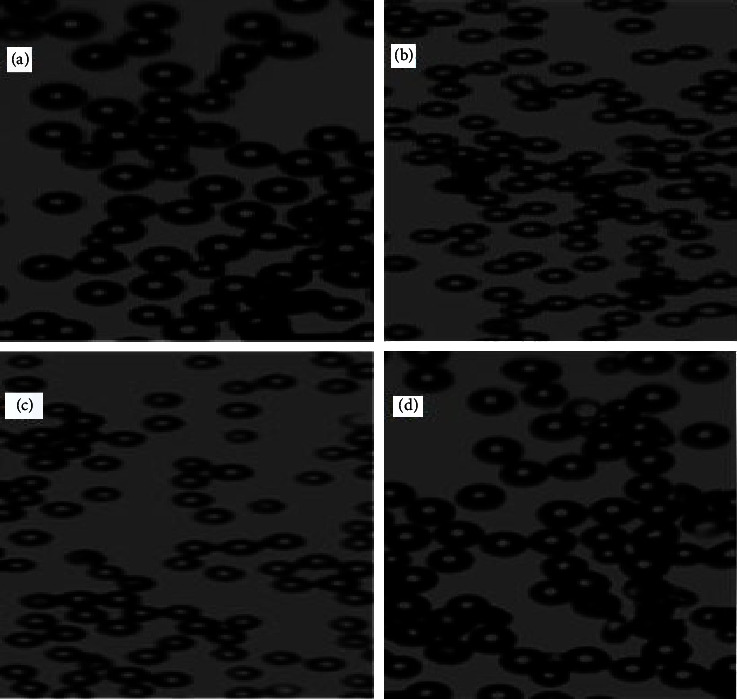
Optical microscopic pictures (40 × 10 magnifications) of the representative microsphere formulations of F1 (a), F3 (b), F4 (c), and F6 (d).

As shown in Figure 6, the optical microscopic results pertaining to the surface morphology and shapes of the microsphere formulations revealed that most of the formulations (F1, F3, F4, and F6) had a regular/spherical shape and were rough in texture. However, F2 and F5 were found to have an irregular or nearly spherical shape.

#### 3.3.2. Particle Size of Microspheres

The average particle size of ibuprofen-loaded LSM microspheres of the different formulations was found to be within the range of 580–710 ± 10 *μ*m (Table 5). Besides, the particle size was observed to increase with the concentration of the polymer.

#### 3.3.3. Density and Related Properties

As mentioned in Table 5, the bulk and tapped densities of LSM microspheres ranged from 0.56 ± 0.04 to 0.57 ± 0.01 g/ml and from 0.65 ± 0.01 to 0.66 ± 0.01 g/ml. Likewise, Carr's index and Hausner's ratio values ranged from 14.05 ± 0.2 to 14.44 ± 0.1 and from 1.16 ± 0.02 to 1.17 ± 0.011, respectively. The angle of repose for all of the formulations ranged from 24.3 ± 0.2 to 27.1 ± 0.2. Thus, all these values confirmed that the prepared microspheres had good flow and compressibility properties.

#### 3.3.4. Drug Content, Percentage Yield, and Entrapment Efficiency

The percentage of drug content encapsulated in LSM microspheres was found to be between 50 ± 0.3 and 84.8 ± 0.4% (Table 6). Among all the formulations, F3 had the highest percentage of drug (84.8 ± 0.4%) followed by F2 (75.2 ± 0.4%) and the least drug content (50 ± 0.3%) was observed for F4. Furthermore, the percentage yield of the formulations was found to be in the range of 82.4 ± 1.0–96.4 ± 0.5% (Table 6). These results indicated that with the increase in the percent yield, the concentration of the polymer also increased. In addition, the percentage of the drug entrapped ranged between 52.08 ± 0.8% and 87.97 ± 0.7% which was significantly higher (*P* < 0.05) than those formulated using the standard polymer. Hence, this signified that the mucilage obtained from LS seed had a higher entrapment efficiency than the standard polymer. In general, % EE was observed to increase with the concentration of the polymer [36].

#### 3.3.5. Swelling Index

The swelling property of the formulations was investigated both in acidic and basic media. The formulations were not able to show any sign of swelling in the acidic media (pH 1.2) even when allowed to stand in the medium for 24 h. However, in the basic medium, the formulations' swelling ability was in the range of 73.6 ± 0.4%–83.6 ± 0.3% (Table 6). Moreover, the swelling index was increased in the basic medium as a function of the concentration of polymer. Thus, as shown in Table 6, LSM microspheres showed a similar percentage of the swelling index as that of the standard polymer (HPMC K100M). Therefore, the data of the swelling index witnessed the ability of the microspheres to obtain swelling at the absorbing surface by absorbing fluids available at the site of absorption (a primary requirement for the initiation of mucoadhesion) [12].

#### 3.3.6. *In-Vitro* Mucoadhesive Property (Wash-Off Test)

As shown in Table 7, an *ex-vivo* mucoadhesive study was conducted using a simulated intestinal fluid, and among the formulations, it was observed that F3 had a significantly higher (*P* < 0.05) mucoadhesive strength by maintaining its adhesion for 18 hr. The results of wash-off tests (Table 7) indicated better mucoadhesive properties of the formulated microspheres over an extended period of time. Moreover, the mucoadhesive strength of the microspheres was found to be directly proportional to the concentration of polymers.

#### 3.3.7. *In-Vitro* Drug Release Profile

**Figure 7:**
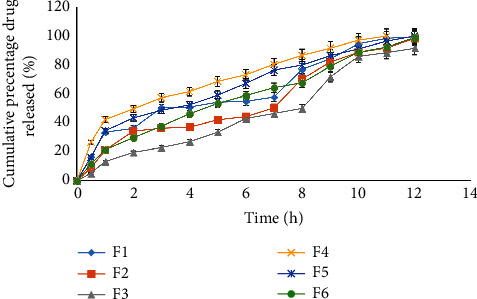
Cumulative percentage drug release profiles of all formulations in PBS (pH 6.8) at 37 ± 0.5°C.

The *In-vitro* drug release profiles of all batches of the microsphere formulations were analyzed in PBS (pH 6.8). Thus, as shown in Figure 7, it was observed that all formulations, except F3 (which released 91.5%) released more than 98.2% of their drug content in 12 hr. Nevertheless, formulations F1, F4, and F5 released 50% of their drug within 6 hr. Whereas, F2 and F3 released 50% of their drug within 7 and 8 hr, respectively. Formulations F1, F4, and F5 released 75% of their content before 8 hr, while F2 and F6 released 75% of their drug content in 9 hr. Thus, F3 showed significant (*P* < 0.05) drug release-sustaining properties than other formulations.

As shown in Figure 7, it was also observed that increasing the concentration of polymers resulted in slower drug release from the mucoadhesive microspheres. Polymer concentration played a major role in controlling the rate of drug release from the microspheres. Thus, the sustained release property of F3 could be attributed to the higher concentration of the polymer, which created thick and swelled polymer gel around the microsphere and became less porous, thus decreasing the rate of drug release. On the other hand, higher polymeric concentration could also increase the diffusion path length around the matrix, which might be a contributing factor to the retarded diffusion of the drugs [37, 38].

**Figure 8:**
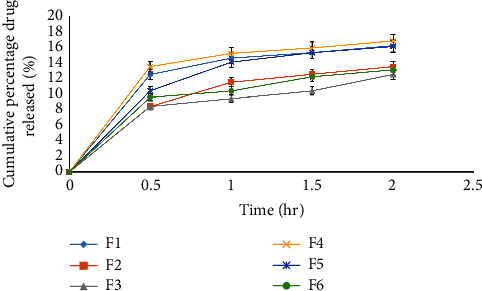
Percentage cumulative drug release profile of all formulations in 0.1 N HCl (pH 1.2) at 37 ± 0.5°C.

The drug release profile of the microsphere formulations was also evaluated in a simulated gastric media of pH 1.2. The cumulative amount of drug released in the first 2 h was found to be from 8.4 ± 0.1 to 16.8 ± 0.1 (Figure 8). The less amount of drug released in the gastric media was probably from the surface of the formulation that was not entrapped within the polymer. Likewise, other studies also reported a negligible amount of drug release from microspheres in a simulated gastric media [36].

#### 3.3.8. *In-Vitro* Drug Release Mechanism and Kinetics

In order to analyze the drug release kinetics and mechanism, the *in-vitro* drug release data of all formulations were fitted to the five common drug release kinetic models. Thus, as shown in Table 8, F1 followed the first-order release kinetics with *n* = 0.5 and F2, F4, and F5 followed the Higuchi model with “*n*” values of 0.65, 0.4, and 0.51, respectively. On the other hand, F3 and F6 followed zero-order drug release kinetics with “*n*” values of 0.85 and 0.64, respectively (Table 8).

Moreover, the accuracy and prediction ability of the models that were compared using a correlation coefficient (*R*^2^) are shown in Table 8. Hence, the *R*^2^ values of the formulations with zero-order drug release kinetics were found to fall in the range of 0.914 to 0.989, which was linearly followed by a fairly linear Korsmeyer–Peppas value ranging from 0.815 to 0.993. Likewise, the release exponent (*n*) values of the Korsmeyer–Peppas that were employed to study the *in-vitro* drug release mechanism of the formulations were observed to be between 0.40 and 0.85. Thus, the formulation F4 followed fickian release with *n* ≤ 0.43, while all the other formulations (F1, F2, F3, F5, and F6) followed non-Fickian drug release mechanism with “*n*” values between 0.43 and 0.85. Hence, this result indicated that the mechanisms of drug release from the microspheres were a combination of diffusion-controlled and erosion-type modeling systems [39].

## 4. Conclusion

Mucoadhesive drug delivery systems are being explored for the localization of the active agents to a particular site. Synthetic and semisynthetic polymers have been playing an important role in designing mucoadhesive drug delivery systems. Mucilage was extracted from the seeds of *Lepidium sativum* Linn., a plant which belongs to the family of *Brassicaceae* and majorly available in the mountainous and alpine regions of East Africa. The LSM mucilage was found to have optimum physicochemical properties (drug entrapment efficiency, *in-vitro* wash-off property, and drug release profile) supporting its mucoadhesive behavior. Microsphere formulations were prepared by following the ionotropic gelation technique using different ratios of LSM and HPMC K100M. All formulations showed excellent *in-vitro* mucoadhesive properties in the simulated goat lumen. The *in-vitro* drug release study confirmed that almost all the formulations sustained release of the drug up to 12 hr. Most of the formulations followed zero-order and Higuchi release kinetics with diffusion and erosion-controlled release mechanism. Thus, this study verified that this mucilage contains important characteristics that make it an ideal alternative to be considered in the case of a sustained drug delivery system and targeted drug delivery system. Besides, this mucilage (as a polymer of the MDDS) can be considered as the best alternative to enhance the bioavailability of poorly soluble drugs and to avoid GIT degradation and first-pass metabolism of some drugs. Therefore, based on the finding of this study, *Lepidium sativum* Linn. mucilage can be considered as a potential alternative polymer for a mucoadhesive drug delivery system.

## Figures and Tables

**Table 1:** Compositions of ibuprofen microsphere formulations prepared in the ratio of 1 : 5, 3 : 5, and 5 : 5.

Ingredients (g)	Formulations					
	F1	F2	F3	F4	F5	F6
Ibuprofen	5	5	5	5	5	5
LSM	1	3	5	—	—	—
HPMC K100M	—	—	—	1	3	5

**Table 2:** Phytochemical screening results of the *Lepidium sativum*. Linn.

Lists of secondary metabolites	Type of test	Result
Alkaloids	Wagner's	+
Carbohydrates	Molisch's	+
Glycosides	Modified Borntrager's	+
Saponins	Froth	−
Phytosterols	Salkowski's	−
Fixed oils and fats	Stain	−
Resins	Acetone-water	−
Phenols	Ferric chloride	−
Flavonoid	Shinoda	+
Tannins	Gelatin	−
Proteins and amino acids	Biuret	+
Coumarin	Con. HCl discoloration	−
Terpenoids and steroids	Liebermann–Burchard	−

**Table 3:** Flow, density, and density-related properties of LSM (*n* = 3 and mean ± SD).

Variables	Values
Bulk density (g/ml)	0.75 ± 0.02
Tapped density (g/ml)	0.69 ± 0.03
Hausner ratio	1.05 ± 0.03
Carr's index (%)	9.98 ± 0.64
Angle of repose (°)	33 ± 0.50
Flow rate (g/sec)	6.32 ± 0.05

**Table 4:** Physicochemical properties of the LSM (*n* = 3 and mean ± SD).

Variables	Values
Loss on drying (% w/w)	8.0 ± 0.2
Swelling index (SI %)	2.9 ± 0.2^*∗*^, 16.5 ± 0.3^*∗∗*^, and 18.8 ± 0.2^*∗∗∗*^
pH	6.2 ± 0.5
Particle size (*μ*m)	182.0 ± 1.2

^*∗*^ = HCl; ^*∗∗*^ = PBS; ^*∗∗∗*^ = water.

**Table 5:** Density and density-related properties of ibuprofen-loaded LSM microspheres (*n* = 3 and mean ± SD).

Formulation	Angle of repose	Flow rate (g/sec)	Bulk density (g/ml)	Tapped density (g/ml)	Carr's index (%)	Hausner's ratio	Particle size (*μ*m)
F1	26.9 ± 0.3	7.13 ± 0.03	0.56 ± 0.04	0.65 ± 0.01	14.44 ± 0.1	1.17 ± 0.01	580 ± 10
F2	26.3 ± 0.2	7.73 ± 0.05	0.56 ± 0.03	0.66 ± 0.01	14.31 ± 0.2	1.17 ± 0.02	650 ± 40
F3	24.5 ± 0.3	7.78 ± 0.04	0.57 ± 0.01	0.66 ± 0.02	14.07 ± 0.3	1.16 ± 0.02	710 ± 8
F4	27.1 ± 0.2	7.21 ± 0.02	0.56 ± 0.01	0.66 ± 0.01	14.35 ± 0.2	1.17 ± 0.01	600 ± 10
F5	26.7 ± 0.1	7.69 ± 0.03	0.57 ± 0.02	0.66 ± 0.01	14.09 ± 0.3	1.16 ± 0.01	650 ± 50
F6	24.3 ± 0.2	7.76 ± 0.02	0.57 ± 0.01	0.66 ± 0.01	14.05 ± 0.2	1.16 ± 0.02	710 ± 10

**Table 6:** Physicochemical characterization of the drug-encapsulated microspheres (*n* = 3 and mean ± SD).

Formulations	Drug-to-polymer ratio	Drug content (g)	Drug content (%)	Yield (%)	Drug entrapment efficiency (%)	Swelling index (%) after 24 h
F1	5 : 1	3.2 ± 0.45	64 ± 0.3	82.4 ± 1.0	66.39 ± 0.1	74.5 ± 0.1
F2	5 : 3	3.76 ± 0.01	75.2 ± 0.4	95.9 ± 0.2	78.5 ± 0.1	79.4 ± 0.5
F3	1 : 1	4.24 ± 0.03	84.8 ± 0.4	96.4 ± 0.5	87.97 ± 0.7	83.6 ± 0.3
F4	5 : 1	2.5 ± 0.04	50 ± 0.3	81.1 ± 0.5	52.08 ± 0.8	73.6 ± 0.4
F5	5 : 3	3.29 ± 0.05	65.8 ± 0.2	95.9 ± 0.3	68.68 ± 0.2	80.6 ± 0.2
F6	1 : 1	3.67 ± 0.03	73.4 ± 0.1	96.3 ± 0.3	76.14 ± 0.4	82.5 ± 0.6

**Table 7:** *In-vitro* mucoadhesive study of the microspheres in PBS of pH 6.8 (*n* = 3 and mean ± SD).

No. of microspheres initially attached	Time (hr)	*% of microsphere formulations remained attached*					
		F1	F2	F3	F4	F5	F6
100	0	100	100	100	100	100	100
	0.5	100	100	100	100	100	100
	1	95.42 ± 1.2	98.41 ± 1.3	99.81 ± 1.8	94.96 ± 1.2	97.49 ± 1.5	98.46 ± 1.6
	2	93.2 ± 2.3	97.29 ± 1.3	99.25 ± 1.2	91.48 ± 2.1	96.81 ± 1.4	98.11 ± 1.8
	3	89.65 ± 1.8	96.38 ± 1.6	98.11 ± 1.5	88.48 ± 1.7	96.11 ± 1.8	97.25 ± 1.5
	4	87.03 ± 1.8	94.38 ± 1.4	97.26 ± 1.1	84.86 ± 1.2	93.89 ± 1.7	96.86 ± 1.3
	5	84.21 ± 2.1	91.67 ± 1.1	95.33 ± 1.4	81.22 ± 1.6	90.84 ± 1.3	95.21 ± 1.1
	6	81.68 ± 2.2	87.12 ± 1.2	92.49 ± 1.3	79.86 ± 1.4	86.98 ± 1.2	91.85 ± 1.5
	7	80.12 ± 1.5	84.33 ± 1.4	90.68 ± 1.1	78.12 ± 1.4	84.13 ± 1.2	90.11 ± 1.6
	8	78.43 ± 1.8	81.12 ± 1.6	88.45 ± 0.5	75.69 ± 0.9	80.28 ± 1.7	87.55 ± 2.1
	9	76.25 ± 1.7	79.67 ± 1.9	85.86 ± 1.1	74.13 ± 1.2	78.49 ± 1.3	85.21 ± 2.1
	10	74.33 ± 1.4	78.45 ± 1.2	81.25 ± 1.3	71.11 ± 1.1	78.21 ± 1.6	80.15 ± 1.2
	11	70.25 ± 1.4	71.15 ± 1.4	78.27 ± 0.8	68.78 ± 1.1	69.48 ± 1.1	77.65 ± 1.4
	12	69.45 ± 1.8	68.11 ± 1.2	74.13 ± 1.6	67.35 ± 1.3	68.67 ± 1.1	73.41 ± 1.3
	18	51.25 ± 1.1	54.31 ± 1.8	61.12 ± 2.1	49.15 ± 0.8	50.41 ± 2.3	55.1 ± 2.5

**Table 8:** Rate constants and correlation coefficients of the drug release kinetic models for all of the six microsphere formulations.

Formulations	Release kinetic models											
	Zero-order		First-order		Higuchi		Hixson–Crowell		Korsmeyer–Peppas		Best fit	Drug release mechanism
	*R* ^2^	K0	*R* ^2^	K1	*R* ^2^	KH	*R* ^2^	KHC	*R* ^2^	*n*		
F1	0.914	−15.910	0.929	−0.122	0.828	−65.31	0.830	0.640	0.815	0.46	First-order	NF
F2	0.929	−14.903	0.713	−0.143	0.943	−64.78	0.878	0.504	0.931	0.65	Higuchi	NF
F3	0.963	−17.784	0.899	−0.207	0.895	−73.95	0.885	0.516	0.966	0.85	Zero-order	NF
F4	0.969	−14.693	0.868	−0.101	0.989	−61.54	0.927	0.663	0.983	0.40	Higuchi	F
F5	0.968	−15.191	0.813	−0.120	0.984	−66.10	0.912	0.636	0.967	0.51	Higuchi	NF
F6	0.989	−16.575	0.865	−0.152	0.981	−64.30	0.919	0.573	0.993	0.64	Zero-order	NF

NF = non-fickian; F = fickian.

## Data Availability

All data pertaining to the findings of this study are on the hands of the principal investigator. Therefore, requests to access data should be made to Hailemichael Embafrash, E-mail: embafrash@gmail.com.
